# Long Non-Coding RNAs at the Chromosomal Risk Loci Identified by Prostate and Breast Cancer GWAS

**DOI:** 10.3390/genes12122028

**Published:** 2021-12-20

**Authors:** Panchadsaram Janaththani, Sri Lakshmi Srinivasan, Jyotsna Batra

**Affiliations:** 1 School of Biomedical Sciences, Faculty of Health, Queensland University of Technology (QUT), Brisbane 4000, Australia; janaththani@gmail.com (P.J.); sri.srinivasan2016@gmail.com (S.L.S.); 2 Translational Research Institute, Queensland University of Technology (QUT), Brisbane 4102, Australia

**Keywords:** long non-coding RNA, prostate cancer, breast cancer, single nucleotide polymorphisms, genome-wide association studies

## Abstract

Long non-coding RNAs (lncRNAs) are emerging as key players in a variety of cellular processes. Deregulation of the lncRNAs has been implicated in prostate and breast cancers. Recently, germline genetic variations associated with cancer risk have been correlated with lncRNA expression and/or function. In addition, single nucleotide polymorphisms (SNPs) at well-characterized cancer-associated lncRNAs have been analyzed for their association with cancer risk. These SNPs may occur within the lncRNA transcripts or spanning regions that may alter the structure, function, and expression of these lncRNA molecules and contribute to cancer progression and may have potential as therapeutic targets for cancer treatment. Additionally, some of these lncRNA have a tissue-specific expression profile, suggesting them as biomarkers for specific cancers. In this review, we highlight some of the cancer risk-associated SNPs that modulated lncRNAs with a potential role in prostate and breast cancers and speculate on how these lncRNAs may contribute to cancer development.

## 1. Introduction

Hormone-related cancers, prostate and breast cancer, accounted for more than 3.6 million newly diagnosed cancer cases worldwide in 2020 [1]. Genetic predisposition has been identified as one of the factors contributing to the risk of these cancers. Genome-wide association studies (GWASs), analyzing common low-penetrance variants, have identified specific risk loci for these cancers [2,3]. Most of the risk-associated single nucleotide polymorphisms (SNPs) identified through GWAS are present in non-protein-coding DNA [4,5,6,7]. This non-coding DNA can regulate the expression of protein-coding genes and maintain the 3D structure of the genome by serving as a scaffold for transcription factors. Alternatively, some non-coding DNA is now found to be transcribed as non-protein-coding RNA (ncRNA) using high-throughput next-generation sequencing platforms [8]. Even though ncRNAs are not translated into proteins, they play a vital part in human complexity from maintaining normal cellular function to playing a broader role in human diseases including cancer [9,10,11,12]. Both ncRNAs and protein machinery involved in the development of diseases have become targets of novel therapeutic approaches [13,14,15]. Based on transcript size, these ncRNAs are grouped into two major classes: small non-coding RNAs (<200 bp) and long non-coding RNAs (lncRNAs) (>200 bp). The small ncRNA class comprises miRNAs, tRNAs, snRNAs, siRNAs, and piRNAs [3,16,17]., LncRNAs have recently been identified as important mediators in many diseases, including cancer [16,18,19]. Long non-coding RNAs (lncRNAs) are RNA transcripts that lack translational potential into functional proteins. The biogenesis of lncRNAs is similar to mRNAs. Most lncRNAs are transcribed by RNA polymerase II while some are also transcribed by RNA polymerase III. Most of the lncRNAs undergo post-transcriptional modifications, such as splicing, polyadenylation, and 5′ capping-like protein-coding RNAs [20]. However, these molecules have several short open reading frames (sORFs) and have very little protein coding potential, which discriminates them from mRNA [21]. Based on their origin, these lncRNAs can be classified as intronic, exonic, intergenic, intragenic, antisense, 3′ and 5′ UTR, promoter-associated (paRNA), and enhancer-associated (eRNA) [22]. Most of the lncRNAs are localized in the nucleus while some are found in both the nucleus and cytoplasm, and some are specifically distributed in the cytoplasm. These lncRNAs play a functional role in gene expression regulation by either cis (targeting genomically local genes) or trans (targeting distant genes) action [22]. Interestingly, recent advanced research has identified several putative coding sORFs suggesting that lncRNAs may be translated into micro peptides with a functional role [23,24,25].

Even though lncRNAs constitute a major part of the human transcriptome, the functional characterization and identification of molecular pathways in which these lncRNAs are involved remains a challenge. Nevertheless, considerable variabilities in the function of lncRNAs can be observed through well-characterized lncRNAs to date. It is evidenced that many lncRNAs are deregulated in prostate and breast cancer and some of their expression has been significantly associated with different stages of cancer. These lncRNAs are proposed to be involved in cancer development by playing functional roles in chromatin remodeling, transcriptional regulation, or post-transcriptional regulation. They show tumor-suppressive or oncogenic potential, emphasizing their potential in targeted therapeutics for prostate and breast cancer [26,27]. In addition, lncRNAs show tissue- and cancer-specific expression patterns, enabling them to be better diagnostic and prognostic tools for cancer therapies [26,28]. Moreover, SNPs could affect the expression and molecular function of lncRNAs, for instance, by disrupting their secondary structure and playing critical roles in tumorigenesis [29]. Recently, there has been increasing evidence in studies linking genetic variants modulating lncRNA expression and prostate or breast cancer risks [6,30]. In this review, we summarize lncRNAs regulated by risk-associated genetic variants (Table 1) in these two hereditary cancers to gain insights into the contribution of lncRNAs to cancer etiology, oncogenic function, and treatment resistance.

## 2. Prostate Cancer Risk-Associated SNPs Modulating lncRNAs

As a multifactorial disease, prostate cancer has several aspects contributing to its etiology, comprising both modifiable and non-modifiable factors [31]. Diet and environmental exposure disruptors, such as bisphenol A, chlordecone, and pesticides [31], are reported as modifiable prostate cancer risk factors. Age is a well-known non-modifiable risk factor for prostate cancer, where the risk of developing cancer increases with age [32,33]. Ethnicity is another non-modifiable contributing factor to the development of prostate cancer, where Asians have been reported to have lower prostate cancer incidence rates than European and American populations [31]. Furthermore, family history and/or heredity is also a known non-modifiable prostate cancer risk factor [33]. There is a considerable amount of evidence for a genetic basis (up to ~57%) contributing to the risk of prostate cancer [34,35]. Recently, a large prostate cancer GWAS identified novel risk loci making it to a total of 269 risk loci to date [36] and the study led to the identification of a genetic risk score of prostate cancer predisposition. Nevertheless, identification of the causal genes has been a major challenge, given the location of a large proportion of these variants are in the non-coding regions. Functional studies are known to complement GWAS results to identify specific genes whose expressions are associated with disease phenotype. One such approach is by expression quantitative trait locus (eQTL), which can identify the association between risk genotype and gene expression, and transcriptome-wide association studies (TWASs), which can assess the association with disease risk throughout the transcriptome. 

One of the few studies to explore prostate cancer GWAS SNPs-associated lncRNAs identified that the prostate cancer-associated SNPs are less polymorphic in the flanking regions, but the SNP density was similar in protein-coding and lncRNA gene regions, indicating the sequences of lncRNA are evolutionarily conserved [37]. This study reported that 52 loci were located within the lncRNA genes, including a new prostate cancer risk-related SNP rs3787016 in a predicted lncRNA *AC1127096.1* [37]. This locus has been initially identified to be associated with prostate cancer risk in members with families having multiple cases of prostate cancer [38]. An independent case-control study in Chinese men reported that the rs3787016 SNP ‘A’ allele was associated with a higher risk of developing prostate cancer in younger individuals as well as individuals with a smoking history or Gleason score ≥7 (4 + 3) or aggressive disease [39]. 

By using a systemic approach for lncRNA based on their position in the promoter region, intercellular functional correlation, eQTL with one or more risk SNPs (cis-eQTL), and differential expression between tumor and normal prostate tissues, Guo et al. shortlisted 45 potential lncRNAs with 50% of prostate cancer risk loci from 122 loci [30]. This included already well-known lncRNAs, such as *KCNQ1OT1*, *H19*, and prostate cancer-associated transcript 1 (*PCAT1).* LncRNA *KCNQ1OT1* was known to act as an miRNA sponge and compete with either miR-211-5p or miR-15 to promote prostate cancer progression [40,41]. Polymorphisms in *H19* were also associated with risk and some of the clinical parameters of bladder cancer [42], hepatocellular carcinoma [43], cervical cancer [42], and urothelial cell carcinoma [44]. Two additional SNPs in *H19*, rs3024270 or rs3741219, were shown to be associated with the risk of perineural invasion of prostate cancer [45]. Increased expression of *H19* was observed in prostate cancer patients with a high Gleason score compared to a low Gleason score and benign prostatic hyperplasia (BPH) [46]. The function of *H19* remains controversial in multiple cancers, including prostate cancer. While knockdown of *H19* in PC3 and DU145 prostate cancer cells reduced cell proliferation and glucose and lactate levels [46], the H19-derived miR-675 axis is described as a suppressor of prostate cancer metastasis, regulating extracellular matrix protein and transforming growth factor β-induced protein (TGFB1) [47]. This suggests that the tumor microenvironment and cell types should be accounted for when determining the functional role of *H19*. In addition, *H19* overexpression increased the expression of stem cell markers *Oct4* and *Sox2* and increased colony formation in the RWPE-1 prostate epithelial cell line [48]. *H19*-dependent transcriptional regulation by estrogen and hypoxia redirected the cells from epithelial to mesenchymal transition (EMT) to β integrin-mediated invasion [49]. 

Guo et al. also reported that of the 45 lncRNAs regulated by non-coding SNPs, 18 lncRNAs’ expression was significantly correlated with 15 prostate cancer risk loci [30]. Moreover, some of these risk SNPs are enriched in the promoter regions of five lncRNAs –*PCAT1*, *RP11-400F19.18*, *RP11-24D8.1*, *RP11-552F3.10*, and *RP11-328M4.2* [30]. This study further identified that the risk SNP rs7463708 in the enhancer region of the *PCAT1* increased the binding of a novel AR interacting partner, ONECUT2, which then looped to the *PCAT1* promoter. Moreover, *PCAT1* was identified as an androgen late-response gene and interacted with AR and lysine-specific demethylase 1 (LSD1) upon prolonged androgen treatment to promote prostate cancer growth [30]. *PCAT1*, which has been identified as the top-ranked lncRNA based on its overexpression in prostate tumor [50], is also known to promote prostate cell proliferation through upregulation of the cMyc protein [51]. Apart from this distal enhancer locus at rs7463708 SNP, another independent risk locus tagged by rs10086908 SNP was associated with *PCAT1* expression, with nine SNPs located across the promoter and exons of the *PCAT1* gene [30]. Moreover, rs1902432 SNP in *PCAT1* was also identified to be associated with an increased risk of PCa [52]. Interestingly, a meta-analysis of five lncRNA polymorphisms in prostate cancer-associated non-coding RNA1 (*PRNCR1*, also known as *PCAT8*) and multiple cancer susceptibility reported that four of the SNPs (rs16901946, rs13252298, rs1016343, and rs1456315) were associated with overall cancer risk while no association was found with rs7007694 SNP [53]. Another small case-control study using 178 prostate cancer patients and 180 BPH cases in the Iranian population identified rs13252298, rs1456315, and rs7841060 SNPs in *PRNCR1* to be associated with prostate cancer risk [54]. There was no significant association between rs7007694 SNP and prostate cancer risk [54], as also reported with overall cancer risk previously [53] and after adjusting for clinicopathological characteristics, such as age, tumor stage, prostate-specific antigen (PSA) levels, Gleason score, perineural invasion, and surgical margin [54]. However, it is important to validate these associations in a larger cohort. *PRNCR1* was reported to have high expression in aggressive prostate cancer and was reported to enhance both ligand-dependent and ligand-independent AR-mediated transcriptional activity by directly binding to the region of 549-623 amino acids of AR and therefore promotes prostate cancer growth [55]. On the contrary, another study by Parolia et al. reported significantly lower expression levels of *PRNCR1* in the prostate cancer models they tested, raising questions about its involvement in AR activation in prostate cancer [56]. Similarly, *PRNCR1* was excluded from the study by Guo et al., due to its undetectable expression in the LNCaP prostate cancer cell line and TCGA prostate adenocarcinoma RNA-sequencing data [30]. In addition to prostate cancer, polymorphisms in *PRNCR1* at the *8q24* locus are also associated with gastric [57], colorectal [58], and lung cancer risk [59], indicating its functional role in multiple cancers. 

A multiethnic meta-analysis study of prostate cancer GWAS in >10 million SNPs in ~80,000 individuals identified a novel prostate cancer risk locus at 9p21 [60]. The prostate cancer risk-associated variant at this locus, rs17694493, is predicted to disrupt the binding motifs of transcription factors STAT1 and RUNX1 and positioned in the intronic region of a novel lncRNA gene *CDKN2B*-*AS1* (also known as *ANRIL*). Moreover, SNPs rs4977574, rs1333048, and rs10757278 in the *ANRIL* gene were also associated with BPH and prostate cancer risk in the Iranian population [61]. Overexpression of *ANRIL* in prostate cancer cells increased cell proliferation and migration by regulating the let-7a/TGFB1/Smad signaling pathway [62], demonstrating the potential molecular mechanism by which this lncRNA mediates cancer progression. 

Prostate cancer risk-associated SNPs, rs11672691 and rs887391, were identified to regulate two *PCAT19* lncRNA isoforms with two distinct transcription start sites, *PCAT19*-short and *PCAT19*-long, through a promoter-to-enhancer switching mechanism [63]. The rs11672691 SNP on chromosome 19 was identified to be associated with both non-aggressive and aggressive prostate cancer risk [64], prostate cancer-specific mortality [65], and poor prognosis after diagnosis [63]. *PCAT19*-long promoted prostate cancer progression by interacting with a nuclear riboprotein, Heterogeneous Nuclear Ribonucleoprotein A/B (HNRNPAB), to upregulate a subset of cell-cycle genes [63], suggesting a novel mechanism for the HNRNPAB role in prostate cancer progression. 

Some SNPs are also found to regulate distant lncRNAs by chromosome looping. For instance, prostate cancer risk SNP rs378854 at the 8q24 locus was found to regulate the expression of lncRNA *PVT1*, which is located 0.5 Mb away from this variant by long-range chromatin looping [66]. Exon 9 of the *PVT1* gene was overexpressed in aggressive prostate cancer cases with African ancestry, suggesting this could be used as a biomarker for metastatic disease [67]. Knockdown of *PVT1* was shown to reduce prostate cancer growth in vitro and in vivo and increase cell apoptosis in prostate cancer cells [68]. Some of these studies are summarized in Table 1.

## 3. Breast Cancer Risk-Associated SNPs Modulating lncRNAs

Breast cancer is the commonly diagnosed cancer in females worldwide. It is a heterogeneous disease on a molecular and clinical level, and has four distinct subtypes: Luminal A, Luminal B, human epidermal growth factor receptor 2 (HER2) overexpression, and triple-negative, based on the status of estrogen receptor (ER), progesterone receptor (PR), and HER2 [69,70]. Breast cancer GWASs have identified more than 200 risk loci, including differential associations with ER+, ER−, or triple-negative breast cancer [7,71,72]. 

A transcriptome-wide association study by Wu et al. identified 26 lncRNAs through eQTL analysis of breast cancer risk loci [73]. The functional role of three of these lncRNAs: *RP11–218M22.1*, *RP11–467J12.4*, and *CTD-3032H12.1*, was confirmed by the significant reduction in cell proliferation on lncRNA knockdown in three breast cancer cell lines, 184A1, MCF7, and T47D, and reduced colony-forming efficiency in MCF7 cells. *RP11–467J12.4*, also known as *PR-lncRNA-1*, is mainly localized in the nucleus, and regulated by P53 in human and mouse cells [74]. LncRNA *CTD-3032H12.1* is predicted to interact with another lncRNA *RP11-20F24.2* and mRNA of *ANKRD30A*, a transcription factor implicated in breast cancer progression, using a tissue-specific co-expression regulatory network model [75]. 

A recent study by Marjaneh et al. exploring multi-exonic non-coding RNA (mencRNA) genes at 139 breast cancer GWAS loci identified more than 4000 mencRNAs using RNA-capture sequencing [76]. Interestingly, the breast cancer risk variants were enriched in the exonic regions of these RNAs, suggesting that these risk variants may impact RNA stability, structure, or function. One example reported in this study for enriched risk variants in exons is the 2q14.2 locus, where three of the four independent risk signals were in the exonic regions of mencRNAs. Furthermore, eQTL analysis shortlisted 800 mencRNAs, including seven signals: XLOC_022678, XLOC_093918, XLOC_112072, XLOC_142280, XLOC_169717, XLOC_195543, and XLOC_209276, overlapping with breast cancer risk signals [76]. Four of these eQTLs were identified to regulate mencRNAs through distal interactions as confirmed by Capture Hi-Seq. This includes the potential causal variants at the estrogen-regulated enhancer of two lncRNAs: *CUPID1* and *CUPID2* at the 11q13 locus, which were previously known to promote homologous-based DNA repair [77]. 

A study carried out by Suvanto et al., analyzing 84 lncRNA and 44 transcribed-ultra conserved RNA (T-UCR, a subtype of lncRNAs) regions, identified SNPs in seven lncRNAs and eight T-UCRs associated with breast cancer risk, which were not previously reported by GWAS studies [6]. This includes risk SNPs, rs71124350, and rs28489579 at the 15q21.1 locus, which correlates with the expression of GA-binding protein transcription factor-β subunit 1 antisense RNA 1 (*GABPB1-AS1*). This lncRNA was predicted to be associated with two miRNA networks: hsa-miR-3613-3p and hsa-miR-7106-5p, which were differentially regulated in breast cancer compared to adjacent normal breast tissues [78]. Although the functional role of *GABPB1-AS1* is not known in breast cancer, it was reported to have a function in other cancers. For instance, *GABPB1-AS1* is known to regulate oxidative stress by regulating translation of its sense protein GABPB1 when exposed to a small molecule compound, Erastin, that induces non-apoptotic iron-dependent oxidative cell death (ferroptosis) in hepatocellular carcinoma (HCC) cells [79]. Moreover, high expression of *GABPB1-AS1* was correlated with better overall survival of HCC patients [79]. Similarly, high *GABPB1-AS1* expression was correlated with better prognosis and inversely correlated with tumor size, TNM stage, and Furhman stage of clear cell renal cell carcinoma patients [80]. This was further validated using in vitro and in vivo assays with *GABPB1-AS1* overexpression models, resulting in reduced proliferation, migration, and invasion in 786-o and caki-1 renal cell cancer cells and reduced tumor growth in xenograft models [80]. 

In addition to these transcriptome-wide lncRNA findings of risk loci, some studies have focused on the risk association of genetic variants in well-known breast cancer-related genes. For instance, rs1899663 and rs7958904 SNPs at lncRNA *HOTAIR*, *HOX* transcript antisense intergenic RNA, were associated with an increased risk of breast cancer in the Southeast Chinese Han population of 969 breast cancer cases and 970 healthy controls [81]. Moreover, rs1899663 SNP was also associated with both disease-free and overall survival in younger cases. On the contrary, Yan et al. reported that rs1899663 and rs4759314 SNPs were associated with reduced breast cancer risk among women with age at menarche >14 while rs920778 SNP was associated with an increased risk in the Chinese population of 502 cases and 504 matched healthy controls [82]. rs1899663 and rs12826786 SNPs were associated with a reduced breast cancer risk in the southeast Iranian population while rs920778 SNP was associated with an increased risk similar to the association reported by Yan et al. [83]. A recent meta-analysis study showed that rs12826786 and rs920778 SNPs at *HOTAIR* were correlated with an increased overall cancer risk [84]. *HOTAIR* belongs to the conserved genomic region of several *HOX* family coding and non-coding genes, and known to play a functional role in embryonic development [85]. Overexpression of *HOTAIR* was correlated with metastasis and poor prognosis of various cancers, including breast cancer [85,86]. Overexpression of *HOTAIR* in breast cancer cells increased invasiveness of these cells in a polycomb repressive complex 2 (PRC2)-dependent manner by reprogramming the polycomb binding profile similar to embryonic fibroblast [87]. Interestingly, *HOTAIR* induction has been shown to also be important for the invasive growth of Claudin-low breast cancer cells, which are triple-negative cancer subtype with low expression of claudin-3, claudinin-4, and claudinin-7 [88]. 

Another well-known cancer-associated lncRNA, *H19*, a maternally inherited imprinted gene, is reported to be overexpressed in breast cancer, and associated with poor prognosis in breast cancer patients, especially in the triple-negative molecular subtype [89,90]. A genetic association study in the Chinese Han population of 464 breast cancer cases and 467 healthy controls did not observe any significant association with breast cancer risk for two SNPs, rs3741219 and rs217727, in the *H19* gene in the overall analysis, but on stratified analysis, rs217727 SNP was found to correlate with breast cancer risk in patients with ER+ or HER2+ or women who had more than two pregnancies [89]. Overexpression of *H19* in breast cancer cells promoted cell proliferation and migration [91] while *H19* knockdown reduced estrogen-induced cell growth of breast cancer cells [92]. However, a meta-analysis study by Mathias et al. analyzing 31 SNPs in 12 lncRNAs could not find any association for these SNPs with breast cancer susceptibility, including rs920778, rs1899663, rs12826786, and rs4759314 SNPs on the *HOTAIR* locus and rs217727, rs3741219, rs2107425, and rs2839698 SNPs on the *H19* locus [93] likely due to the smaller number of studies included for this analysis. This emphasizes the need for comprehensive functional analysis with experimental evidence to improve our understanding of how these genetic variants contribute to breast cancer pathology (Table 1).

**Table 1:** LncRNAs associated with risk SNPs in prostate and breast cancer.

	lncRNA	SNP	Function of lncRNA	References
Prostate cancer	*PCAT1*	rs7463708	Promote prostate cancer growth by interacting with AR and LSD1 upon prolonged androgen treatment, Promote prostate cancer cell proliferation through c-Myc upregulation	[30,50,51]
		rs10086908		
		rs1902432		
	*PRNCR1/PCAT8*	rs16901946	Enhance ligand-dependent and -independent AR-mediated transcriptional activity	[53,54,55]
		rs13252298		
		rs1016343		
		rs1456315		
	*CDKN2B-AS1/ANRIL*	rs4977574	Increase prostate cancer cell proliferation and migration by modulating the let-7a/TGFB1/Smad signaling pathway	[60,61,62]
		rs1333048		
		rs10757278		
	*PCAT19*	rs11672691	*PCAT19*-long isoform promotes prostate cancer progression by upregulating a subset of cell-cycle genes via interaction with HNRNPAB	[63,64,65]
		rs887391		
	*PVT1*	rs378854	*PVT1* knockdown inhibits prostate cancer growth in vitro and in vivo and increased cell apoptosis	[66,68]
Breast cancer	*RP11–218M22.1*	rs12422552	Knockdown reduced breast cancer cell proliferation and colony formation	[73]
	*RP11–467J12.4/* *PR-lncRNA1*	rs3112612	Knockdown reduced breast cancer cell proliferation and colony formation	[73]
	*CTD-3032H12.1*	rs28539243	Knockdown reduced breast cancer cell proliferation and colony formation, predicted to interact with lncRNA RP11-20F24.2 and mRNA of ANKRD30A	[73,74]
	*GABPB1-AS1*	rs71124350	Predicted association with two miRNA networks, which are differentially regulated in breast cancer	[6,78]
		rs28489579		
	*HOTAIR*	rs1899663	Overexpression of *HOTAIR* correlates with metastasis of breast cancer, increase breast cancer cell invasiveness via reprogramming PRC2 binding	[81,82,83,84,85,86,87]
		rs7958904		
		rs4759314		
		rs920778		
		rs12826786		
	*H19*	rs3741219	Overexpression of *H19* promoted breast cancer cell proliferation and migration and knockdown reduced estrogen-induced breast cancer cell growth	[89,90,91]
		rs217727		

## 4. Conclusions

Recently, there has been remarkable progress in our understanding of the multifaceted role of lncRNAs and the genetic variants impacting lncRNA expression and function, recognizing them as critical players in prostate and breast cancer progression. Although a majority of these cancer risk-associated genetic variants are found in non-coding RNA loci, only a few studies have focused on uncovering the role of these SNPs in modulating the structure and function of lncRNAs in cancer progression. Emerging sequencing techniques and bioinformatic analysis are helpful in predicting the putative function of the lncRNA. Databases, such as lncRNASNP2 [94] and LincSNP 3.0 [95], provide information on how these SNPs modulate the lncRNA structure and function. Some of these lncRNAs are differentially expressed in disease progression models and cancer subtypes, highlighting their potential to be used as a diagnostic and prognostic biomarker. For example, PCA3 (also known as DD3) is the only FDA-approved lncRNA used for prostate cancer diagnosis [96], which is overexpressed in prostate tumors compared to non-malignant tissues. Nevertheless, using lncRNA-SNPs to predict the disease progression and/or therapeutic options is still in an early stage, since how these SNPs regulate the expression or function of lncRNAs remains uncertain. Most of these studies have identified cancer-associated lncRNAs through their expression correlation with the SNP genotype. Moreover, whether these SNPs are true causal SNPs or correlated variants in high linkage disequilibrium with the causal SNPs still needs to be clarified. Advancing techniques including CRISPR genome editing may provide comprehensive insights in this field to help identify the functional role of the cancer-associated risk variants through lncRNAs in disease progression and identify their applicability as novel therapeutic targets or biomarkers for multiple cancers.

## Data Availability

Not applicable.
